# Supplementary material for: Enhanced Expression of Human Endogenous Retroviruses, TRIM28 and SETDB1 in Autism Spectrum Disorder
Source: Int J Mol Sci. 2022 May 25;23(11):5964. doi: 10.3390/ijms23115964 (PMC9180946; doi:10.3390/ijms23115964)
Supplement: Supplementary file 1 [file ijms-23-05964-s001.zip › ijms-1705116-supplementary.pdf]

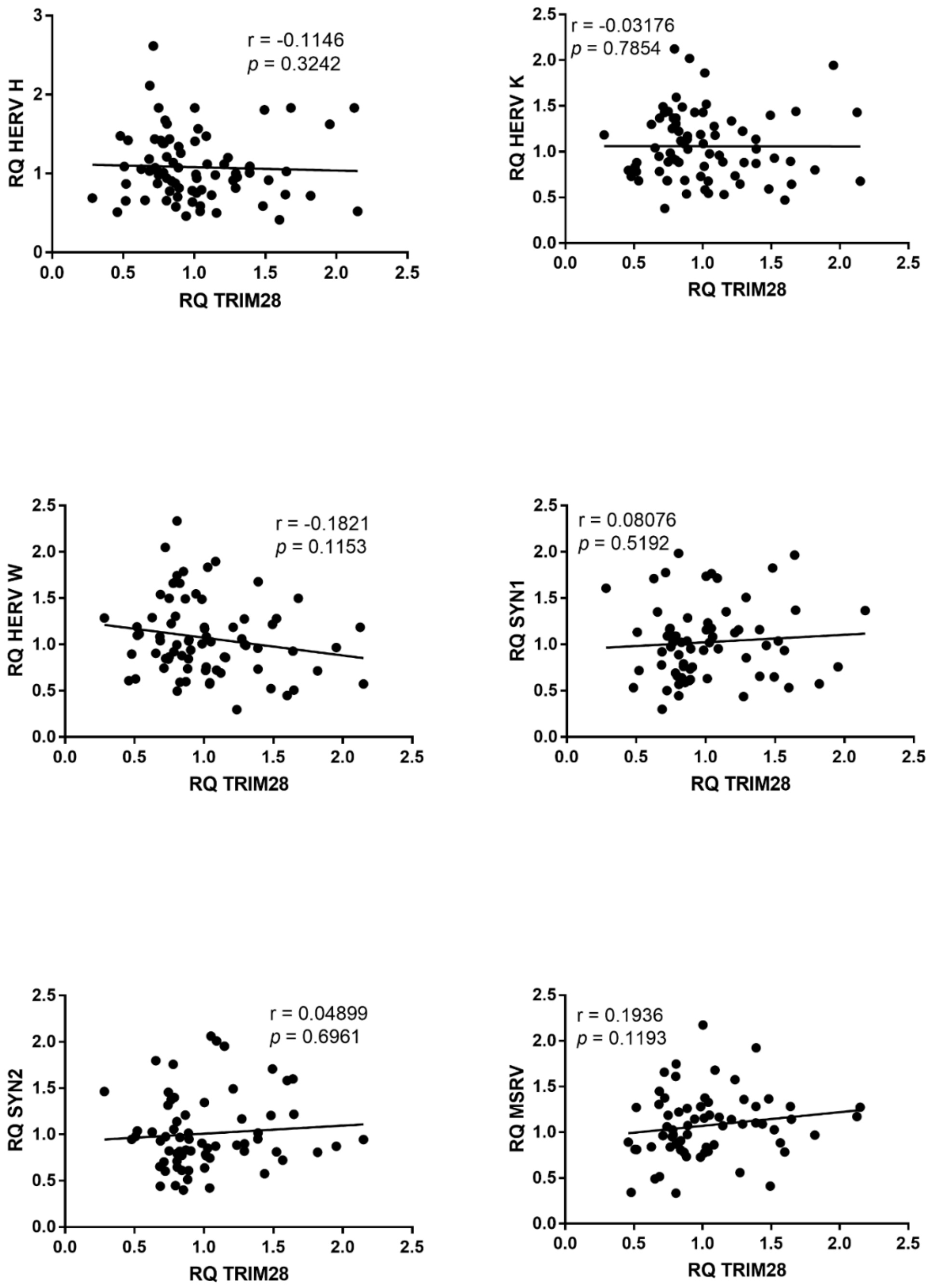

Figure S1. Correlations between transcription levels of TRIM28 and HERV sequences in whole blood from 79 healthy children (HC).

RQ: Relative Quantification. Circles show the mean of three individual measurements. Line: Linear regression line. Statistical analysis: Spearman correlation test.

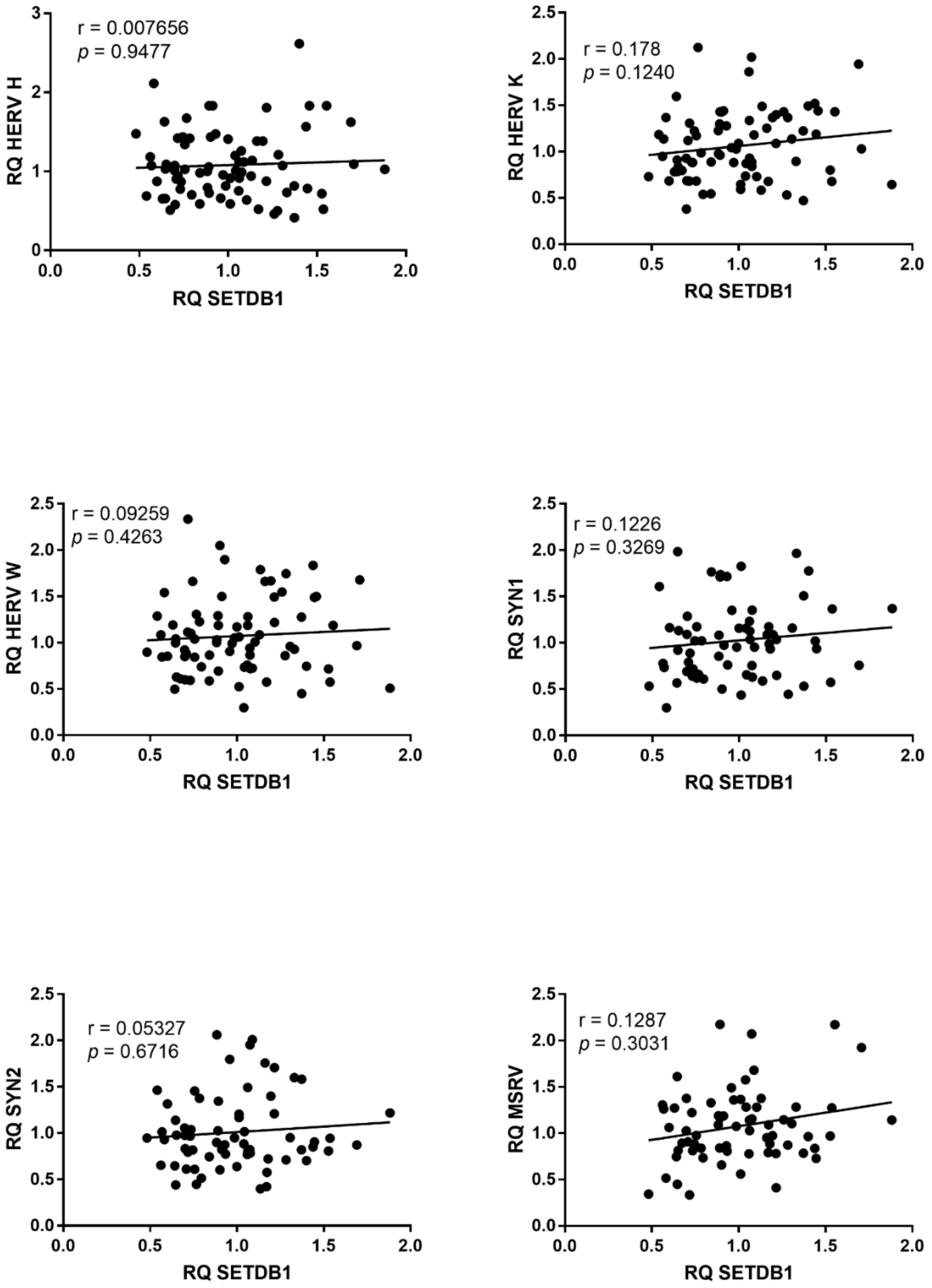

Figure S2. Correlations between transcription levels of SETDB1 and HERV sequences in whole blood from 79 healthy children (HC).

RQ: Relative Quantification. Circles show the mean of three individual measurements. Line: Linear regression line. Statistical analysis: Spearman correlation test.
